# Identification of key phosphorylation sites in PTH1R that determine arrestin3 binding and fine-tune receptor signaling

**DOI:** 10.1042/BCJ20160740

**Published:** 2016-11-10

**Authors:** Diana Zindel, Sandra Engel, Andrew R. Bottrill, Jean-Philippe Pin, Laurent Prézeau, Andrew B. Tobin, Moritz Bünemann, Cornelius Krasel, Adrian J. Butcher

**Affiliations:** 1 Institut für Pharmakologie und Klinische Pharmazie, Philipps-Universität Marburg, 35043 Marburg, Germany; 2 Institut de Génomique Fonctionnelle (IGF), University of Montpellier, CNRS UMR5203, 34094 Montpellier, France; 3 INSERM U1091, 34094 Montpellier, France; 4 The Protein and Nucleic Acid Chemistry Laboratory, University of Leicester, Hodgkin Building, Lancaster Road, Leicester LE1 9HN, U.K.; 5 Institute of Molecular, Cell and Systems Biology, University of Glasgow, Glasgow G12 8QQ, Scotland, U.K.; 6 MRC Toxicology Unit, University of Leicester, Leicester LE2 2RG, U.K.

**Keywords:** arrestin, G-protein-coupled receptor (GPCR), parathyroid hormone receptor 1 (PTH1R), phosphorylation

## Abstract

The parathyroid hormone receptor 1 (PTH1R) is a member of family B of G-protein-coupled receptors (GPCRs), predominantly expressed in bone and kidney where it modulates extracellular Ca^2+^ homeostasis and bone turnover. It is well established that phosphorylation of GPCRs constitutes a key event in regulating receptor function by promoting arrestin recruitment and coupling to G-protein-independent signaling pathways. Mapping phosphorylation sites on PTH1R would provide insights into how phosphorylation at specific sites regulates cell signaling responses and also open the possibility of developing therapeutic agents that could target specific receptor functions. Here, we have used mass spectrometry to identify nine sites of phosphorylation in the C-terminal tail of PTH1R. Mutational analysis revealed identified two clusters of serine and threonine residues (Ser489–Ser495 and Ser501–Thr506) specifically responsible for the majority of PTH(1–34)-induced receptor phosphorylation. Mutation of these residues to alanine did not affect negatively on the ability of the receptor to couple to G-proteins or activate extracellular-signal-regulated kinase 1/2. Using fluorescence resonance energy transfer and bioluminescence resonance energy transfer to monitor PTH(1–34)-induced interaction of PTH1R with arrestin3, we show that the first cluster Ser489–Ser495 and the second cluster Ser501–Thr506 operated in concert to mediate both the efficacy and potency of ligand-induced arrestin3 recruitment. We further demonstrate that Ser503 and Thr504 in the second cluster are responsible for 70% of arrestin3 recruitment and are key determinants for interaction of arrestin with the receptor. Our data are consistent with the hypothesis that the pattern of C-terminal tail phosphorylation on PTH1R may determine the signaling outcome following receptor activation.

## Introduction

The parathyroid hormone receptor 1 (PTH1R) is a member of family B of G-protein-coupled receptors (GPCRs), which includes the glucagon receptor and the calcitonin receptor [1]. The main function of PTH1R is in bone and kidney where it mediates extracellular Ca^2+^ homeostasis and bone turnover [2,3]. In addition to these functions, PTH1R has been identified in the intestinal mucosa and in several carcinomas and adenocarcinomas [4]; however, its role here is currently less well understood. The endogenous ligand for the PTH1R is parathyroid hormone (PTH), which is released from the parathyroid gland and is composed of 84 amino acids, of which the first 34 amino acids mediate its biological activity [5]. In bone, PTH mediates both anabolic and catabolic processes. Clinical studies have demonstrated that intermittent administration of PTH(1–34) mediates osteoanabolic effects through increased proliferation and differentiation of osteoblasts [6], and PTH or its biological N-terminal fragment PTH(1–34) is currently in use as a therapy for osteoporosis. Continuous treatment with PTH(1–34), however, is associated with increased bone resorption through osteoblast-mediated activation of osteoclasts. PTH, PTH-related peptide and cytokines, such as interleukin (IL)-6 or IL-11, lead to receptor-mediated expression of receptor activator of nuclear factor κ-B ligand (also known as tumor necrosis factor-related activation-induced cytokine), which in turn promotes maturation and activation of bone-degrading osteoclasts [7].

The cellular signaling profile of PTH1R is complex but well understood [8]. Activation of PTH1R with low concentrations of agonist results in a rapid increase in cyclic adenosine monophosphate (cAMP) mediated through coupling to G_s_ and activation of adenylate cyclase [9], whereas agonist concentrations in the micromolar range mediate phospholipase C (PLC) activation and IP_3_ accumulation and the release of calcium from intracellular stores. This is considered to be particularly relevant when local PTH concentrations are high, as is the case in the epiphyseal plate during longitudinal growth of long bones [10]. The switch from G_s_ to G_q_ signaling is believed to be achieved through binding of the Na^+^/H^+^ exchanger regulatory factor 2 (NHERF2), which simultaneously associates with PLC and a PDZ domain in the PTH1R C-terminus [11]. In addition, activation of the Gα_12/13_-phospholipase D/RhoA and mitogen-activated protein kinase (MAPK) pathways through PTH1R have been reported [12,13].

Like the majority of class A and class B GPCRs, the PTH1R becomes rapidly phosphorylated in the C-terminal tail in an agonist-dependent manner [14–16]. It has been demonstrated that Ser473, Ser475 and Ser491 in the PTH1R C-terminus are substrates for cAMP-dependent protein kinase A (PKA). Protein kinase C (PKC) phosphorylation was also observed in the C-terminal tail of PTH1R, but the sites involved could not be precisely localized [15]. Pharmacological inhibition of PKA or mutation of putative PKA phosphorylation sites does not appear to affect substantially agonist-regulated receptor phosphorylation [15], and it seems therefore likely that that the majority of agonist-regulated receptor phosphorylation in the C-terminal tail of PTH1R is mediated by other kinases such as GRKs [14–16]. It is known that PTH1R activation results in robust recruitment of arrestin and that the interaction of arrestin with the involves C-terminal tail phosphorylation. However, it has previously been reported that a PTH1R C-terminally truncated after residue 474 was still able to recruit arrestin in a ligand-dependent manner, albeit to a lesser extent than wild-type (WT) [17]. Interaction of arrestin3 with the PTH1R is reportedly facilitated by overexpression of NHERF by forming a ternary complex with arrestin3 and the PTH1R [18]. Previously published data suggest that, in addition to its canonical role in receptor desensitization, PTH1R–arrestin interaction can promote sustained cAMP formation in a manner dependent on extracellular-signal-regulated kinase 1/2 (ERK1/2) [19,20].

It is now clear that the function of arrestin is more than simply to serve as an accessory protein for receptor endocytosis and as such has been shown to play an important role in complex cellular signaling pathways and physiological functions [21]. Although it is clear that GPCR phosphorylation regulates arrestin recruitment, the precise mechanisms which couple PTH1R phosphorylation with arrestin signaling are currently not known. It has been suggested that the pattern of receptor phosphorylation may represent a ‘barcode’ that may, to some extent, direct arrestin-dependent signaling in response to the phosphorylation profile of the receptor [22] To date, the PTH1R phosphorylation profile or the possibility that PTH1R signaling through arrestin may be directed in a phosphorylation-dependent manner has not been investigated.

In the current study, we have mapped the phosphorylation sites in the C-tail of PTH1R using mass spectrometry and by employing a series of PTH1R C-tail mutants. Furthermore, we have characterized the functional impact of these sites with respect to arrestin recruitment and the temporal stability of arrestin–receptor complexes. Our comprehensive analysis reveals multiple phosphorylation sites in the PTH1R C-tail and the existence of two phosphorylation clusters that are crucial for high-affinity arrestin binding. Our data provide evidence for the existence of a mechanism by which PTH1R phosphorylation could modulate the affinity and stability of arrestin–receptor complexes and thereby affect on PTH1R signaling.

## Materials and methods

Unless otherwise stated, all biochemical and reagents were from Sigma (Taufkirchen, Germany).

### Plasmids and mutagenesis

The HA–PTH1R [23] was subcloned into pcDNA3 (ThermoFisher, Karlsruhe, Germany). The yellow fluorescent protein (YFP)-labeled PTHR was constructed by replacing the stop codon by an XbaI restriction site using a cytomegalovirus (CMV) forward primer and the reverse 5′-TCTAGACATGACTGTCTCCCACTCTTC-3′ primer. The PCR product was inserted before the coding sequence for eYFP. Mutations were inserted into the PTH1R using the QuikChange® protocol (Stratagene, Santa Clara, CA, USA). To construct the truncated PTH1R, a CMV forward primer was used combined with a reverse primer (5′-AAAAAATCTAGATGCCAGTGTCCAGCG-3′) inserting a stop codon and an XbaI restriction site after Ala480. All constructs were verified by sequencing. Arrestin3–cyan fluorescent protein (CFP) has been described previously [24].

### Enzyme-linked immunosorbent assay (ELISA) for quantification of receptor expression on the cell surface

Relative receptor expression levels were determined as described previously [25]. Briefly, human embryonic kidney (HEK)293 cells transfected with the indicated HA–PTHR constructs were fixed in 4% paraformaldehyde (PFA), blocked with 1% fetal bovine serum (FBS) in PBS and incubated with a horseradish peroxidase (HRP)-labeled anti-HA antibody (Roche, Boulogne-Billancourt, France) at 1:2000 dilution for 30 min. An HRP substrate was added and chemoluminescence was measured on a plate reader (Infinite® 200, Tecan, Lyon, France).

### Transient expression in HEK293T cells or stable expression in HEK293 cells

HEK293T cells were maintained in Dulbecco's modified Eagle's medium (DMEM) supplemented with 10% FBS, penicillin (50 mg/ml) and streptomycin (50 mg/ml). Stable and transient transfections were performed using Effectene® (Qiagen, Hilden, Germany) or Lipofectamine 2000® (Thermo Fisher) following the manufacturer's protocol. An HEK293 cell line stably transfected with the human HA–PTH1R was generated by standard procedures using G418 (500 µg/ml) for selection.

### PTH1R purification and mass spectrometry

HEK293 cells stably expressing the HA–PTH1R were grown to 80% confluency in 150 mm dishes. For receptor purification, cells from 30 dishes were resuspended in Krebs/HEPES buffer containing 0.1% BSA and stimulated with 1 µM PTH(1–34) (Bachem, Bubendorf, Switzerland) for 8 min. Membranes were then prepared and solubilized as described recently [26]. Briefly, solubilized receptors were immunoprecipitated using anti-HA affinity matrix and separated by SDS/PAGE. After Coomassie blue staining, receptors were excised from the gel, washed with 100 mM triethylammonium bicarbonate followed by reduction of disulfide bonds with 10 mM dithiothreitol and alkylation of cysteines using 100 mM iodoacetamide. Purified receptors were digested with 1 µg of sequencing grade trypsin (Promega, Southampton, UK) overnight at 37°C. Tryptic phosphopeptides were enriched using immobilized metal affinity chromatography using PHOS-select™ affinity resin. Eluted proteins were analyzed by LC–tandem mass spectrometry (MS/MS) using an LTQ Orbitrap mass spectrometer (Thermo Fisher Scientific, Rockford, IL, USA) as described recently [26].

### [^32^P]orthophosphate labeling and PTH1R immunoprecipitation

[^32^P]Orthophosphate labeling, agonist incubation, receptor solubilization, immunoprecipitation and autoradiography were performed as described recently [27]. Briefly, HEK293 cells stably expressing HA–PTH1Rs were grown in six-well plates. Cells were preincubated for 60 min in phosphate-free Krebs buffer containing 100 µCi/ml [^32^P]orthophosphate (PerkinElmer, Billerica, MA, USA) before being stimulated for 5 min with 1 µM PTH(1–34). Cells were immediately lysed by the addition of 0.5 ml of buffer containing 20 mM Tris, 150 mM NaCl, 3 mM EDTA, 1% Nonidet P-40 and 0.25% sodium deoxycholate, pH 7.4. Receptors were immunoprecipitated using anti-HA affinity matrix (Roche, Mannheim, Germany). Immunoprecipitated proteins were resolved using gel electrophoresis on 8% SDS gels and visualized by autoradiography. A portion of the immunoprecipitated material was retained, resolved using gel electrophoresis on 8% SDS gels, transferred onto a PVDF membrane and immunoblotted with anti-HA antibody (Roche, Mannheim, Germany) for the detection of total receptors.

### PTH1R–arrestin3 interaction assays

A bioluminescence resonance energy transfer (BRET) assay was used to monitor interactions between PTH1R and arrestin3. C-terminally YFP-tagged PTH1R WT or PTH1R mutants as described above were co-transfected with arrestin3–*Renilla* luciferase (Rluc) at a ratio of 4:1 using Lipofectamine 2000 (Life Technologies, Invitrogen, Gran Island, NY, USA) according to the manufacturer's instructions After 24 h, cells were subcultured into poly-d-lysine-coated white 96-well microplates and incubated for a further 24 h prior to the assay. Cells were then washed with Hanks balanced salt solution and incubated in this buffer for 30 min prior to conducting the assay. To begin the assay, the Rluc substrate coelenterazine h (Life Technologies, Invitrogen, Gran Island, NY, USA) was added to a final concentration of 2.5 µM and incubated for 10 min at 37°C before PTH(1–34) was added. Following a further 5 min incubation, luminescence emissions at 535 and 475 nm were measured using a CLARIOstar (BMGLabtech, Offenburg, Germany), and the BRET signal was presented as the 535/475 ratio multiplied by 1000 to yield the arbitrary milli-BRET units.

### Microscopic fluorescence resonance energy transfer measurements and data evaluation

Dynamics of arrestin–receptor interaction were performed on an inverted fluorescence microscope (IX71, Olympus, Hamburg, Germany). Single cells plated on poly-d-lysine-coated glass coverslips were observed using a ×100 oil-immersion objective (UPlanSApo 100×/1.40 oil, Olympus). YFP was excited with a laser at 491 nm; CFP was excited at 405 nm. An optosplit II (Cairn Research, Faversham, UK) was used to split YFP and CFP (T495lpxr, Chroma, Olching, Germany). To minimize photobleaching, the illumination frequency was set to 0.2 Hz. For CFP detection, an ET470/40× filter and, for YFP detection, an ET535/30 filter (Chroma) were used. The signal was amplified by a charge-coupled device (CCD) camera (ImagEM, Hamamatsu, Herrsching, Germany). Fluorescence resonance energy transfer (FRET) was calculated by *F*_YFP_/*F*_CFP_, and traces were corrected for spillover of CFP into the YFP channel as well as for YFP direct excitation. Individual FRET recordings were averaged and shown as absolute alterations in FRET normalized to baseline. Quantification of relative expression levels of fluorescently labeled proteins was performed as described recently [27].

### Dual-color fluorescence recovery after photobleaching microscopy

Fluorescence recovery after photobleaching (FRAP) was performed as described previously [27,28] with minor modifications. HEK293T cells transiently expressing arrestin3–CFP and N-terminally HA-tagged PTHRs labeled with YFP at the C-terminus were treated with a monoclonal anti-HA antibody (HA.11 16B12, BioLegend) followed by 30 min incubation with a polyclonal anti-mouse antibody at 37°C before being stimulated with 100 nM PTH(1–34) in 0.1% BSA/Tyrode's buffer. The scan speed was set to 100 Hz, the image format was 256 × 256 pixels and the zoom factor was set to 6.0.

### ERK1/2 activation and cAMP measurements by homogeneous time-resolved fluorescence (HTRF)

As previously reported [25,29], measurement of ERK1/2 activation and cAMP accumulation in HEK293 cells was performed using the Cellul'Erk immunoassay kit and the cAMP Dynamics 2 competitive immunoassay kit (Cisbio Bioassay). For ERK1/2 activation, HEK293 cells were transiently transfected with HA-tagged PTH1R WT, the PD1, PD2 or the PD3 mutant along with arrestin3 using Lipofectamine® 2000 according to the manufacturer's protocol. Cells were serum-starved for at least 18 h prior to stimulation with 500 nM PTH(1–34) in serum-free DMEM containing 0.1% BSA. The cell lysates were transferred to 384-well plates, and both an Eu^3+^- cryptate-labeled anti-ERK monoclonal and a d2-labeled anti-phospho-ERK monoclonal antibody were added. The readout was performed after 2 h using a PHERAstar plate reader (BMG Labtech, Champigny s/Marne, France). For cAMP measurements, HEK293 cells were transfected with HA–PTH1R WT or the PD3 mutant as described above. Prior to lysis, cells were stimulated for 15 min with the indicated concentration of PTH(1–34) in DMEM containing 0.1% BSA and 100 µM isobutylmethylxanthine (IBMX). The cell lysates were transferred to 384-well plates, and cAMP-cryptate antibodies and d2-labeled cAMP were added. After 1 h of incubation, measurements were performed using a PHERAstar plate reader.

### Data analysis

Concentration–response relationships were analyzed by three-parameter non-linear regression using GraphPad Prism 5.0 software (San Diego, CA, USA). For statistical tests, where only two datasets were being compared, unpaired Student's *t*-test (two-tailed) was used, where *P* < 0.05 was deemed statistically significant. Where more than two datasets were compared, one- or two-way analysis of variance (ANOVA) tests were used with *P* < 0.05 being accepted as significantly different. ANOVA tests were followed by Bonferroni's *post hoc* test, Dunnett's multiple comparison test or Dunn's multiple comparison test. All statistical analyses were performed using GraphPad Prism 5.0 or GraphPad 4.0 software. For BRET titration-binding experiments, a one-site binding equation was fitted.

## Results

### Identification of phosphorylation sites in PTH1R

**Figure 1.**
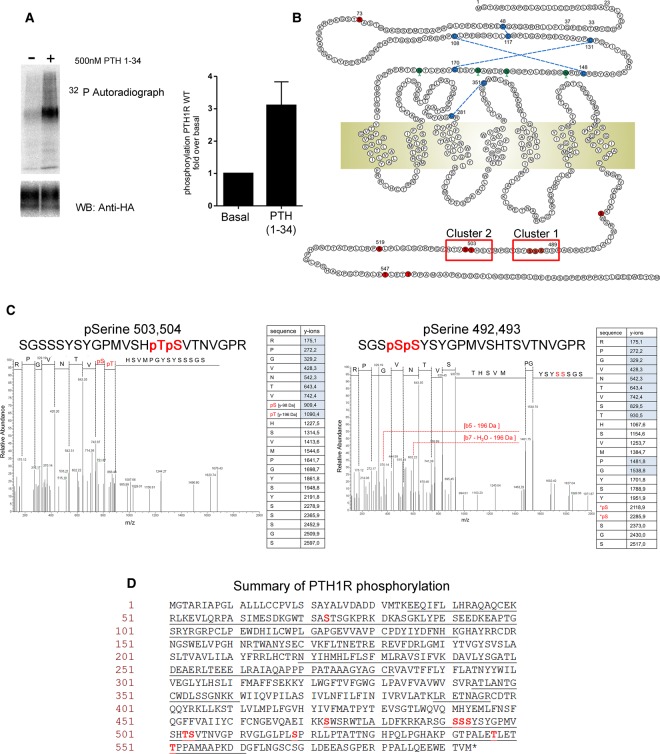
Mass spectrometry identifies phosphorylation sites in PTH1R. (**A**) HEK293T cells were transiently transfected with HA-tagged PTH1R and labeled with [^32^P]orthophosphate followed by immunoprecipitation. HEK293 cells stably transfected with PTH1R–HA were used to immunoprecipitate PTH1R, which was then digested with trypsin and analyzed by mass spectrometry. For ^32^P labeling and mass spectrometry studies, cells were stimulated with 500 nM PTH(1–34) for 8 min. Left panel: autoradiograph and Western blot (anti-HA antibody) loading control. Right panel: levels of ^32^P were quantified by densitometry and are presented as fold increases in phosphorylation relative to non-stimulated controls. Data are representative of three independent experiments* *± SEM. (**B**) Schematic representation of PTH1R. Locations of phosphorylation sites identified by MS/MS are indicated in red, and red boxes indicate the positions of phosphorylation site clusters 1 and 2. (**C**) Representative MS/MS spectra and associated fragmentation tables for two PTH1R phosphopeptides are shown. (**D**) Primary amino acid sequence of PTH1R indicating in red the amino acids identified as being phosphorylated and underlined in black are the regions of PTH1R covered by the analysis.

**Figure 2.**
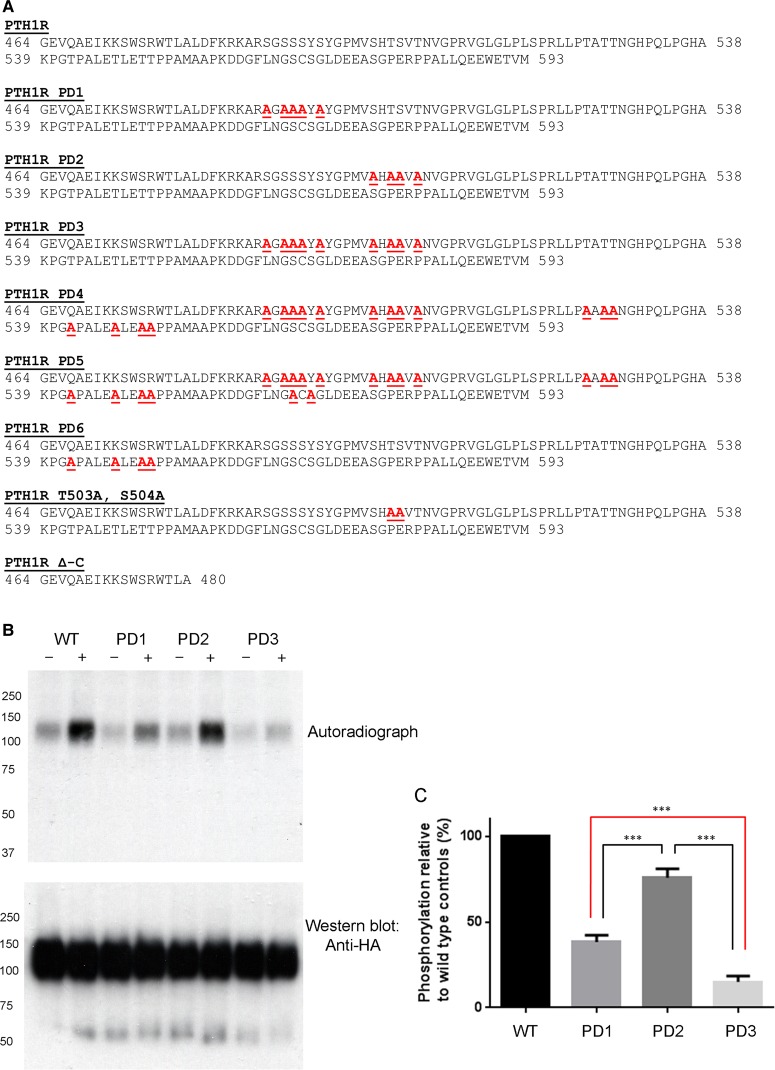
Mutational analysis of PTH1R reveals regions of agonist-regulated receptor phosphorylation. (**A**) Amino acid sequences of the C-terminal tail regions of the PTH1R mutants used in the present study. Positions of serine and threonine residues that have been changes to alanine are indicated in red and underlined. (**B**) Upper panel: [^32^P]orthophosphate labeling studies were performed on HEK293T cells transiently expressing HA-tagged PTH1R WT, PD1, PD2 or PD3 mutants. Cells were stimulated with 1 µM PTH(1–34) for 8 min before immunoprecipitation and exposure for autoradiography. Lower panel: Western blots using anti-HA antibodies were performed to demonstrate equal gel loading. (**C**) Autoradiographs from (**B**) were quantified using densitometry and presented as ^32^P incorporation relative to PTH1R WT controls. Representative of four independent experiments ± SD. ***P < 0.001 determined by ANOVA followed by Tukey's multiple comparison test.

The human PTH1R showed a robust increase in agonist-mediated phosphorylation with a 3.0 ± 1.3-fold increase following stimulation with 500 nM PTH(1–34) when monitored using [^32^P]orthophosphate labeling (Figure 1A). To identify the precise phosphorylation sites within the PTH1R, a mass spectrometry-based study was conducted on tryptic peptides generated from immuno-purified PTH1R following stimulation with 1 µM PTH(1–34). LC–MS/MS analysis of enriched PTH1R tryptic phosphopeptides identified ten phosphorylation sites in total; of these, nine were located in the C-terminal tail (S473, S491, S492, S493, T503, S504, S519, T547 and T551; Figure 1B,C; Table 1). These sites were largely arranged within two distinct clusters. These consisted of ‘cluster 1’ containing phosphorylation sites within region S489–S495 and ‘cluster 2’ that contained sites within S501–T506 (Figures 1C and 2A).

The mass spectrometry methods used provided excellent coverage of the PTH1R C-terminus with 22 of 26 potential phosphorylation sites being identified within tryptic peptides (Figure 1D). It is therefore likely that most of the phosphorylation events associated with the C-terminal tail of the PTH1R were identified in the present study. In contrast, there was only 30% coverage of the short cytoplasmic third intracellular loop. No phosphorylation sites were identified in this region, but because of the low coverage of this region three potential phosphorylation sites (two threonine and one serine) were not observed and it is not possible therefore to conclude that there is no phosphorylation within the third intracellular loop. Interestingly, LC–MS/MS sequencing revealed phosphorylation of Ser73 in the extracellular N-terminus of the PTH1R (Figure 1B). Phosphorylation of a GPCR within an extracellular domain has not previously been reported.

### Differential contribution of serine/threonine clusters to total PTH1R phosphorylation

**Figure 3.**
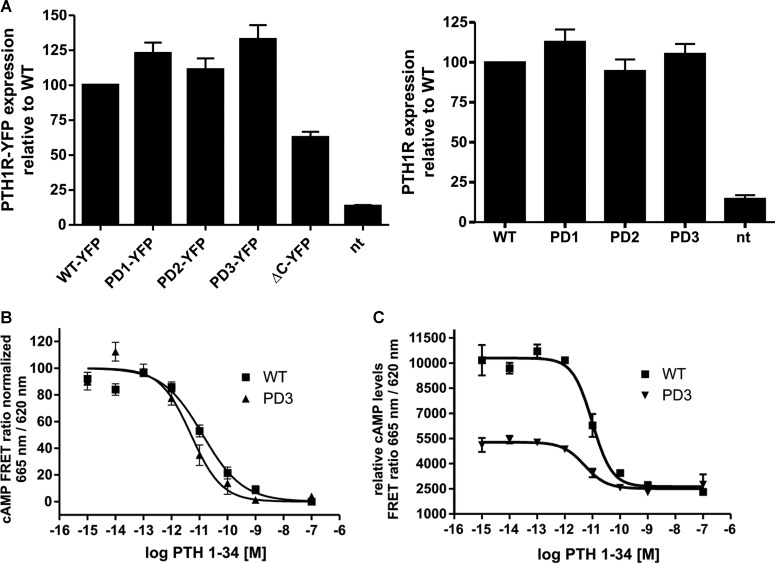
Mutations within the C-terminal tail of PTH1R do not adversely affect receptor expression or G-protein coupling. (**A**) Cell-surface expression of the indicated receptors was assayed by transiently transfecting the HA-tagged receptors into HEK293T cells. Cell-surface ELISAs were performed using an HRP-conjugated anti-HA antibody to detect cell-surface receptor expression in non-permeabilized cells. Data are normalized to PTH1R WT expression levels. Mean values of three independent experiments ± SEM are shown. (**B**) HEK293T cells were transfected with PTH1R WT or mutant PD3 and arrestin3, and concentration–response curves were generated for PTH(1–34) to stimulate cAMP production. Mean values from three independent experiments normalized to the maximum response for each construct are shown. (**C**) Raw FRET values from data generated in (**B**).

Since the distribution of phosphorylation sites within the proximal C-terminus of PTH1R appeared to be arranged in two distinct clusters, mutant receptors were engineered where serine or threonine amino acids within each of the clusters were mutated to alanine residues (Figure 2A). Mutating all of the serine/threonine (S/T) residues to alanine within cluster 1 generated a mutant termed PD1, whereas replacing the S/T residues in cluster 2 generated the mutant PD2. Combining mutations of clusters 1 and 2 generated mutant PD3 (Figure 2A). Importantly, the cell-surface expression of WT PTH1R and mutant receptors (PD1–3) appeared similar (Figure 3A). [^32^P]Orthophosphate labeling of HEK293T cells transiently expressing PD1, PD2 or PD3 followed by receptor immunoprecipitation revealed that the S/T residues within clusters 1 and 2 did not contribute equally to receptor phosphorylation (Figure 2B). Thus, upon stimulation with 1 µM PTH(1–34), ^32^P incorporation into mutant PD1 was significantly reduced by 61.6 ± 3.99% compared with WT. In contrast, ^32^P incorporation into PD2 was reduced by only 24.9 ± 5.1% compared with WT. Mutation of both clusters in mutant PD3 reduced agonist-mediated phosphorylation by 84.9 ± 4.3% compared with WT, almost completely eliminating agonist-mediated receptor phosphorylation. These results demonstrate that although the mass spectrometry data indicate that there are additional phosphorylation sites in the C-terminal tail that are outside of cluster 1 and cluster 2 (Figure 1B,D), the majority of agonist-mediated phosphorylation occurs within these two clusters.

To further investigate the effect of the C-tail mutations on PTH1R receptor function, concentration–response studies for PTH(1–34)-induced cAMP accumulation were carried out with the WT PTH1R and the more extensively mutated PD3 mutant. We observed a similar sensitivity to PTH(1–34) for the WT and for the PD3 mutant (pEC_50_ values of 10.88 ± 0.08 and 11.3 ± 0.1, respectively) with no significant change in the Hill slope of the agonist curve and similar maximal cAMP responses (Figure 3B,C and Table 2). Although we observed that the PD3 mutant displayed a higher constitutive activity compared with the WT receptor (Figure 3C), these data indicate that the extensively mutated PD3 is not compromised in its ability to couple with G-proteins.[Table BCJ-2016-0740TB1]

**Table 1 BCJ-2016-0740TB1:** Unique PTH1R phosphopeptides identified by mass spectrometry Phosphorylated amino acids are underlined, also shown are the peptide locations, the Mascot ion score and the number of times the peptide was observed. Asterisks denote amino acid residues upon which the precise position of phosphorylation could not be determined.

Peptide sequence	Localization	Mascot ion score	Delta (ppm)	Numbers of observations
**GWTSApSTSGKPR**	68–79	53.8	0.22	1
**pSWSRWTLALDKR**	473–485	35.8	−2.2	1
**SGpSSSYSYGPMVSHTSVTNVGPR**	489–511	45.8	6.2	>30
SGS*pS*pSYSYGPMVSHTSVTNVGPR	489–511	60.0	−1.9	>20
**SGSSSYSYGPMVSHpTpSVTNVGPR**	489–511	38.1	−2.8	5
**VGLGLPLpSPR**	511–521	61.4	−2.2	>20
**PGTPALEpTLETTPPAMAAPK**	540–559	44.5	−1.4	1
**PGTPALETLETpTPPAMAAPK**	540–559	52.9	−1.7	5

**Table 2:** Potency (pEC_50_) and Hill slope values of PTH(1–34) at PTH1R WT and PTH1R PD3 mutant receptors in cAMP assays Values represent the means ± SEM from three independent experiments.

	PTH1R WT	PTH1R PD3
pEC_50_ ± SEM	10.88 ± 0.08317	11.3 ± 0.1090
Hill slope ± SEM	0.6083 ± 0.06337	0.7885 ± 0.1382

### Functional impact of PTH1R C-tail phosphorylation sites on arrestin recruitment

**Figure 4.**
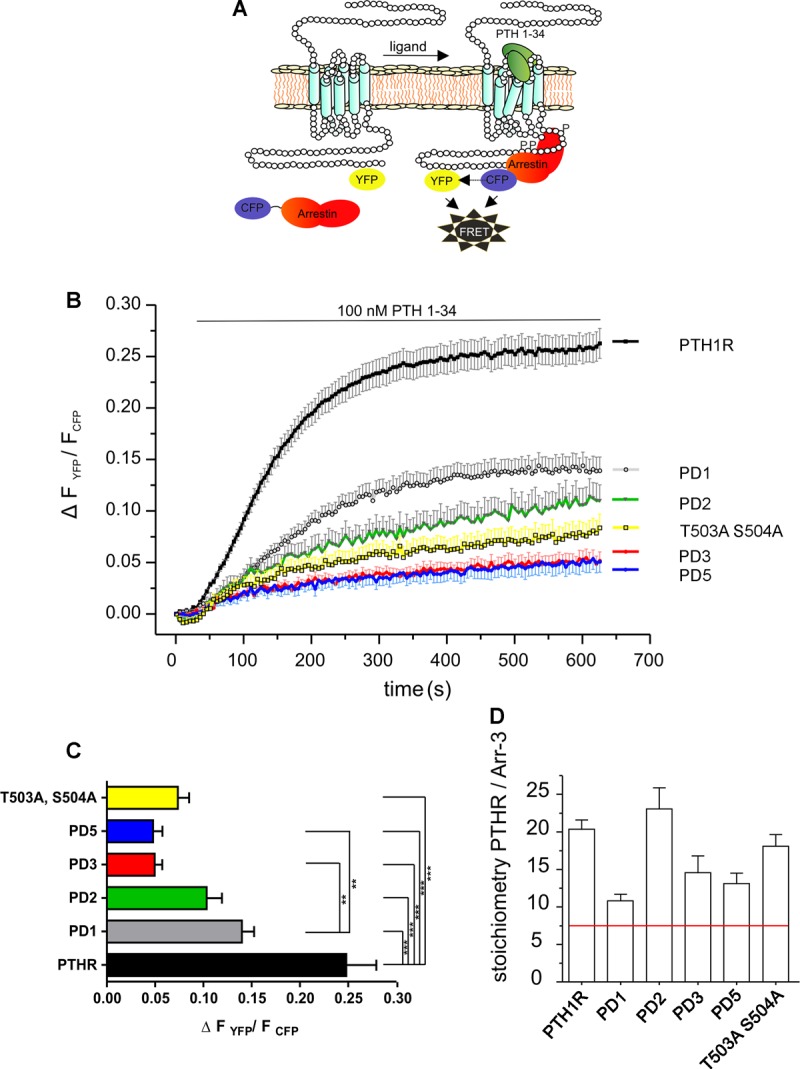
FRET approach to monitor PTH1R–arrestin3 interactions. (**A**) Schematic representation of PTH1R and arrestin3 during FRET assays. (**B**) HEK293T cells were transiently transfected with PTH1R, PD1, PD2, PD3, PD5 or T503A, S504A constructs that were C-terminally labeled with eYFP, and arrestin3–CFP was also transfected. Agonist-dependent FRET changes were monitored following stimulation with 100 nM PTH(1–34) for the times indicated. Individual recordings were averaged (mean ± SEM — PTH1R *n* = 11, PTH1R PD1 *n* = 10, PTH1R PD2 *n* = 10, PTH1R PD3 *n* = 18, PTH1R PD5 *n* = 12 and PTH1R T503A, S504A *n* = 12). For clearer visualization, mean curves are displayed with SEM of Δ*F*_YFP_/*F*_CFP_ in one direction. (**C**) Determination of absolute FRET changes in each measurement. Statistics were performed by the Kruskal–Wallis test followed by Dunn's multiple comparison test. **P < 0.01; ***P < 0.001. (**D**) Relative expression levels of PTH1R eYFP and arrestin3–CFP. The red line indicates a 1:1 stoichiometry of YFP/CFP as determined by a reference construct. Excess acceptor fluorophore-labeled receptors were confirmed in all experiments.

Previous studies have shown that the opossum PTH1R interacts robustly with arrestin2 and arrestin3 in a ligand-dependent manner [17,30]. Our recent studies on the M_3_-muscarinic receptor [31], GHSR1a [32] and GPR120 [26], had indicated that distinct phosphorylation sites, or patterns of receptor phosphorylation, could mediate differential signaling outcomes, including differences in receptor–arrestin interaction. Hence, we examined whether phosphorylation at clusters 1 and 2 of the PTH1R contributed equally to receptor/arrestin interaction. The interaction between YFP-tagged PTH1R C-tail mutants and arrestin3 was characterized using FRET in single living HEK293T cells transiently transfected with CFP-tagged arrestin3 (Figure 4A). In this system, the WT PTH1R showed a robust increase of 24.8 ± 12.7% in FRET after stimulation with 100 nM PTH(1–34) (Figure 4B). Subsequent analysis of PTH1R PD1, PD2 and PD3 revealed that PD1 mutant interaction with arrestin was reduced by 42.1 ± 10.7% in comparison with the WT receptor (Figure 4B), indicating that phosphorylation within cluster 1 did make a contribution to arrestin recruitment. Interestingly, mutation of phosphorylation sites in the mutant PD2 had a greater impact on arrestin interaction, decreasing the recruitment of arrestin by up to 60% despite having only a relatively small impact on receptor phosphorylation compared with PD1. Hence, it would seem that although cluster 1 contributing most to agonist-regulated receptor phosphorylation, it is phosphorylation sites located within cluster 2 that contribute more to the regulation of arrestin recruitment.

Mutant PD3 in which both cluster 1 and cluster 2 phosphorylation sites are mutated showed an additional decrease in arrestin recruitment. Removal of nine additional potential phosphorylation sites in PTH1R in a mutant termed PD5 showed no further reduction in the recruitment of arrestin compared with PD3, indicating that phosphorylation of residues outside clusters 1 and 2 did not make a significant contribution to arrestin3 recruitment.

### Importance of Thr503 and Ser504 in cluster 2 for arrestin recruitment

To characterize the key residues within cluster 2 responsible for determining arrestin binding, a mutant in which only one serine and one threonine within the cluster are mutated to alanine (PTH1R T503A, S504A) was generated. The FRET change from this mutant was found to be 7.0 ± 14.2%, which corresponds to a 72% signal loss of the amplitude achieved with the WT PTH1R (Figure 4B,C). Hence, these data identify Thr503 and Ser504 within the second cluster as key determinants of arrestin binding. Differences in agonist-induced FRET amplitudes could potentially be due to alterations in the acceptor/donor expression if the donor expression exceeds acceptor expression. Expression levels were therefore determined, and it was confirmed that, in all measurements, the proteins carrying the acceptor fluorophore were overexpressed compared with the donor fluorophore-tagged proteins (Figure 4D).

### Confirmation of the contribution of clusters 1 and 2 to arrestin interaction with PTH1R using a BRET-based recruitment assay

**Figure 5.**
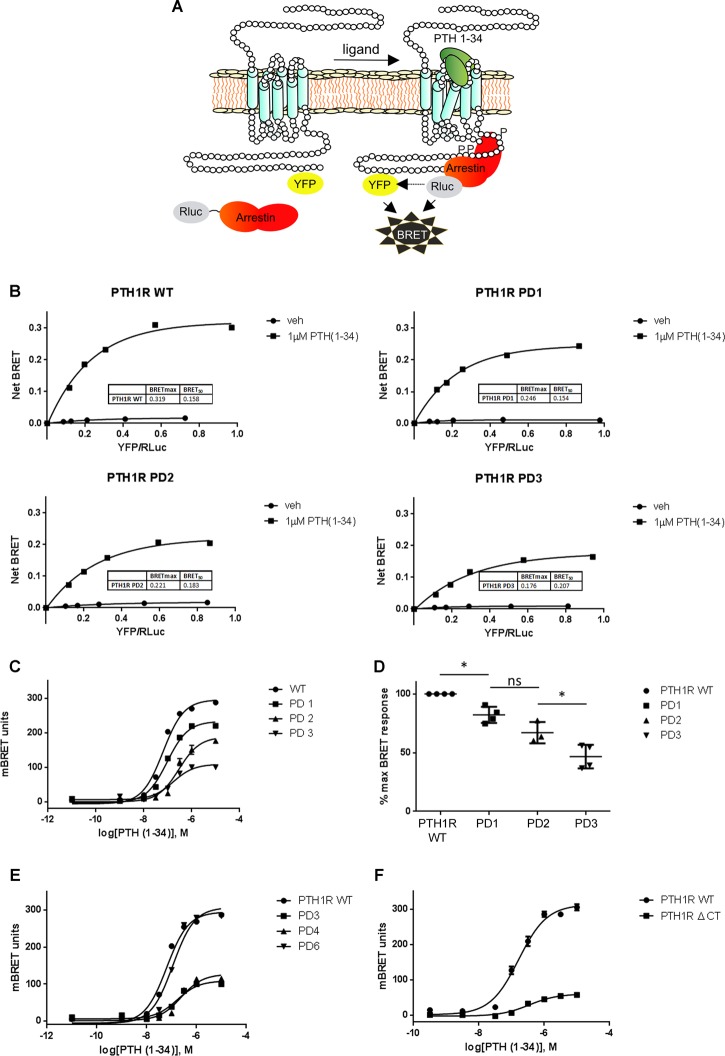
BRET approach to monitor PTH1R–arrestin3 interaction. (**A**) Schematic representation of PTH1R and arrestin3 constructs used during BRET measurements. (**B**) HEK293 cells were transiently transfected with constant amounts of arrestin3–Rluc and increasing amounts of PTH1R, PD1, PD2 or PD3 constructs C-terminally labeled with eYFP. Net BRET induced by stimulation with 1 μM PTH(1–34) for each construct is expressed as a function of the donor/acceptor ratio. BRET_max_ and BRET_50_ values are shown for each construct. (**C**) Concentration–response curves were generated for PTH(1–34) to promote PTH1R interaction with arrestin3. (**D**) Maximum BRET responses for each construct are displayed as a percentage of the response from PTH1R WT, and values are representative of three independent experiments ± SEM. Differences between maximal signal for the individual mutants were significant as indicated, *P < 0.05. (**E** and **F**) Concentration–response curves for PTH(1–34) to promote the recruitment of arrestin3 to PTH1R C-terminal tail mutants PD4, PD6 and PTH1R Δ-C are displayed.

To further evaluate the effects of C-tail phosphorylation sites on the interaction of arrestin with the PTH1R, a BRET–arrestin interaction assay was employed (Figure 5A). Donor/acceptor titration experiments were performed in which HEK293 cells were transfected with a fixed amount of arrestin3–Rluc (the BRET donor) and increasing amounts of WT PTH1R or C-tail mutants fused to eYFP (BRET acceptors). Both WT PTH1R and mutants showed a robust increase in PTH(1–34)-induced BRET, which appeared to be saturated at donor/acceptor ratios greater than 0.6 (Figure 5B). From these experiments, the donor/acceptor ratio at which half-maximum BRET was observed can be calculated. Single exponential fits were made to the curves, and from these, the BRET_max_ and BRET_50_ values were determined. The BRET_50_ values for PD2 and PD3 were both higher than those for WT PTH1R, suggesting a reduction in the affinity between arrestin and the receptor. Interestingly, the BRET_50_ value for PD1 was similar to that for PTH1R WT, suggesting a similar affinity for this interaction despite a reduction in the maximum binding (Figure 5B). Concentration–response curves were generated for arrestin3 recruitment by PTH1R and mutants in response to increasing concentrations of PTH(1–34) (Figure 5C). The maximum binding for PD1 was reduced to 80.8 ± 4.4% compared with the WT PTH1R. In contrast, PD2 was reduced to 66.9 ± 5.2% compared with WT and PD3 was reduced to 46.6 ± 5.0% (Figure 5C,D). There was also a small decrease in potency for PD2 (pEC_50_ = 6.55 ± 0.009) and PD3 (pEC_50_ = 6.75 ± 0.01) compared with WT (pEC_50_ = 7.16 ± 0.03). Analysis of additional mutants in which both phosphorylation site clusters remain intact and other adjacent residues are mutated, termed PD6, or in which PD1 and PD2 are removed and nine additional mutations are introduced, termed PD5, indicated that mutation of sites outside clusters 1 and 2 have a minimal additional impact on arrestin3 recruitment (Figure 5E). Taken together, these data confirm the importance of clusters 1 and 2 in the recruitment of arrestin to PTH1R and also indicate that cluster 2 may contribute more to arrestin recruitment and affinity than cluster 1.

### Phosphorylation within clusters 1 and 2 contributes to, but is not essential for, arrestin recruitment to PTHR1

Mutation of serine and threonine residues within clusters 1 and 2, to generate mutant PD3, resulted in a mutant PTH1R, which showed substantial decreases in both basal and agonist-mediated phosphorylation and a substantial reduction in its ability to recruit arrestin3. However, arrestin3 binding was not completely eliminated when measured by either FRET or BRET methods. To investigate whether additional determinants of arrestin recruitment remained within the C-terminal tail of PTH1R, a mutant receptor was generated in which the entire C-terminal tail was removed leaving the receptor truncated shortly after the putative end of helix VIII. This receptor mutant was found to both express and traffic to the plasma membrane, although expression levels appeared to be reduced compared with the WT PTH1R when assayed by cell-surface (ELISA) (Figure 3A). Interestingly, this PTH1R mutant was still able to recruit arrestin3 in an agonist-dependent manner albeit with much reduced efficacy (20% compared with WT PTH1R; Figure 5F), suggesting that there are determinants outside the C-tail, including clusters 1 and 2, which are able to contribute to arrestin3 recruitment.

### Phosphorylation within cluster 2 is important for the high-affinity interaction of arrestin with PTH1 receptors

**Figure 6.**
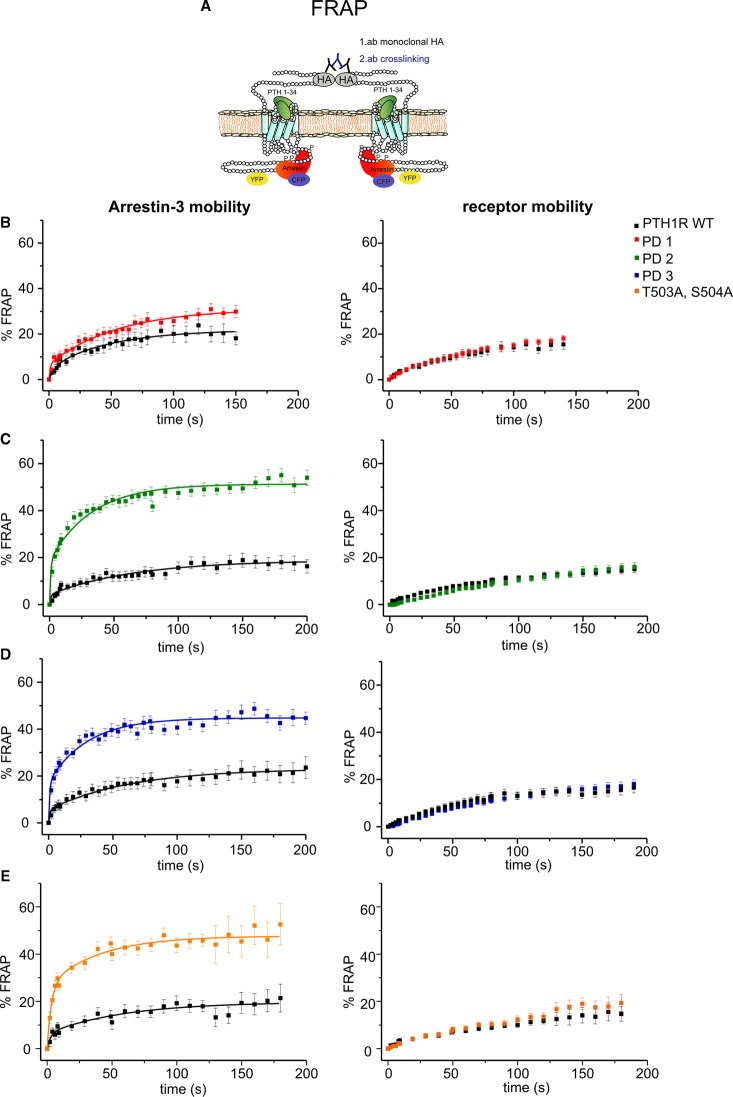
FRAP approach to monitor arrestin affinity toward activated receptors. (**A**) Schematic representation of immobilized PTH1R–arrestin3 complexes during FRAP experiments. (**B–E**) HEK293T cells were transiently transfected with receptors that were both N-terminally HA-tagged and C-terminally YFP-tagged and with arrestin3–CFP. Cells were preincubated for 30 min with a monoclonal anti-HA antibody, followed by 30 min of incubation with a polyclonal anti-mouse antibody at 37°C before being stimulated with 100 nM PTH(1–34). Mobility of CFP-labeled arrestin3 and YFP-labeled receptors was monitored simultaneously after photobleaching of a small spot and the equatorial plane of the plasma membrane. Left panel: mean percentage arrestin3–CFP recovery ± SEM at the PD1 receptor (*n* = 24; WT control *n* = 15), the PD2 mutant (*n* = 20; WT control *n*= 21), the PD3 mutant (*n* = 21; WT control *n* = 21) or the T503A, S504A mutant (*n* = 17; WT control *n* = 15). Right panel: recovery of YFP-labeled receptors ± SEM. Statistics are described in Table 3.

The FRET and BRET experiments shown above demonstrate that phosphorylation sites within the region cluster 2 are important determinants for arrestin recruitment to PTH1R (Figure 4). As PTH(1–34) dissociates very slowly from activated PTH1Rs, it is difficult to determine whether arrestin recruitment to PTH1R or receptor mutants is dependent on its dissociation from activated receptors. Thus, to study whether the removal of phospho-acceptor clusters in the PTH1R C-tail changes the temporal stability of activated arrestin–receptor complexes at the plasma membrane, we used an assay based on dual-color FRAP. This technique has previously been used to monitor the steady-state affinity of arrestin3 to agonist-bound receptors in single living cells [27]. In these experiments, HA-tagged PTH1R–YFP receptors were cross-linked at the plasma membrane and, therefore, the lateral mobility of arrestin–receptor complexes was reduced. After photobleaching, the extent and the kinetics of arrestin3 recovery represent the release of arrestin3 from immobilized activated receptors followed by subsequent rebinding of free arrestin3. To exclude the possibility that the observed differences in arrestin mobility resulted from insufficiently cross-linked receptors, we ensured that basal receptor mobility was ≤30% (it was 20% on average). WT PTH1R stimulated with PTH(1–34) showed arrestin recovery, which was reduced to levels similar to that of the receptor after antibody cross-linking (Figure 6B), indicating low levels of mobility and a high-affinity interaction. In contrast with FRET experiments in which the receptor mutant PD1 reduced the FRET amplitude by 45% (Figure 4), but in good agreement with the BRET experiments (Figure 5B), the influence of PD1 on arrestin affinity in the FRAP experiments appeared limited as the speed and extent of arrestin3 recovery was comparable between the WT PTH1R and the PD1 mutant (Table 3). In contrast, phosphorylation sites within cluster 2 appeared to play an important role not only for arrestin recruitment but also for arrestin affinity as the mobile arrestin fraction at the PD2 mutant was significantly higher than that at the WT receptor (Figure 6C and Table 3). No additional increase in the arrestin3 exchange rate was observed in the PD3 mutant (Figure 6D and Table 3), suggesting that the residues between Ser501 and Thr506 are key determinants for the high-affinity arrestin interaction with PTH1R.

**Table 3:** Kinetics of arrestin3 interaction with PTH1R WT and receptor mutants measured by FRAP Values represent the means ± SEM from three independent experiments. The kinetic parameter *k*_slow_ was obtained by biexponential curve fitting. *t*_0.5_ values were obtained by calculating ln(2)/*k*.

FRAP	Arr-3 recovery ± SEM (%)	*k*_slow_ ± SEM (s^−1^)	Slow half-life (s)
PTH1R WT	19.07 ± 0.68–23.94 ± 0.86	0.0167 ± 0.010–0.014 ± 0.002	40.7–49.5
PTH1R PD1	30.9 ± 2.5	0.019 ± 0.0025	36.5
PTH1R PD2	51.2 ± 0.71	0.031 ± 0.0036	22.4
PTH1R PD3	44.9 ± 0.67	0.033 ± 0.0043	21
PTH1R T503A, S504A	47.7 ± 2.2	0.025 ± 0.0111	27.7

**Table 4:** Statistical values for PTH(1–34)-induced pERK1/2 activation in PTH1R WT, PD1, PD2 and PD3 mutants

Dunnett's multiple comparison test	*P*-value
WT vs. PD1 5 min	>0.05
WT vs. PD2 5 min	<0.05
WT vs. PD3 5 min	<0.01
WT vs. PD1 30 min	>0.05
WT vs. PD2 30 min	<0.05
WT vs. PD3 30 min	<0.01
WT vs. PD1 60 min	>0.05
WT vs. PD2 60 min	>0.05
WT vs. PD3 60 min	>0.05
WT vs. PD1 90 min	>0.05
WT vs. PD2 90 min	>0.05
WT vs. PD3 90 min	<0.01

To further investigate the roles of residues within PD2, the PTH1R T503A, S504A mutant was analyzed. Interestingly, and in agreement with FRET data, this mutant displayed reduced arrestin affinity (Figure 6E); the mobile arrestin fraction was increased 2-fold compared with the WT receptor and the exchange rate was comparable with that of the PD2 or PD3 mutants (Table 3), again suggesting that these two residues are key determinants for arrestin recruitment and high-affinity binding.

### PTH1R C-terminal phosphorylation sites regulate agonist-dependent co-internalization of arrestin with PTH1Rs

**Figure 7.**
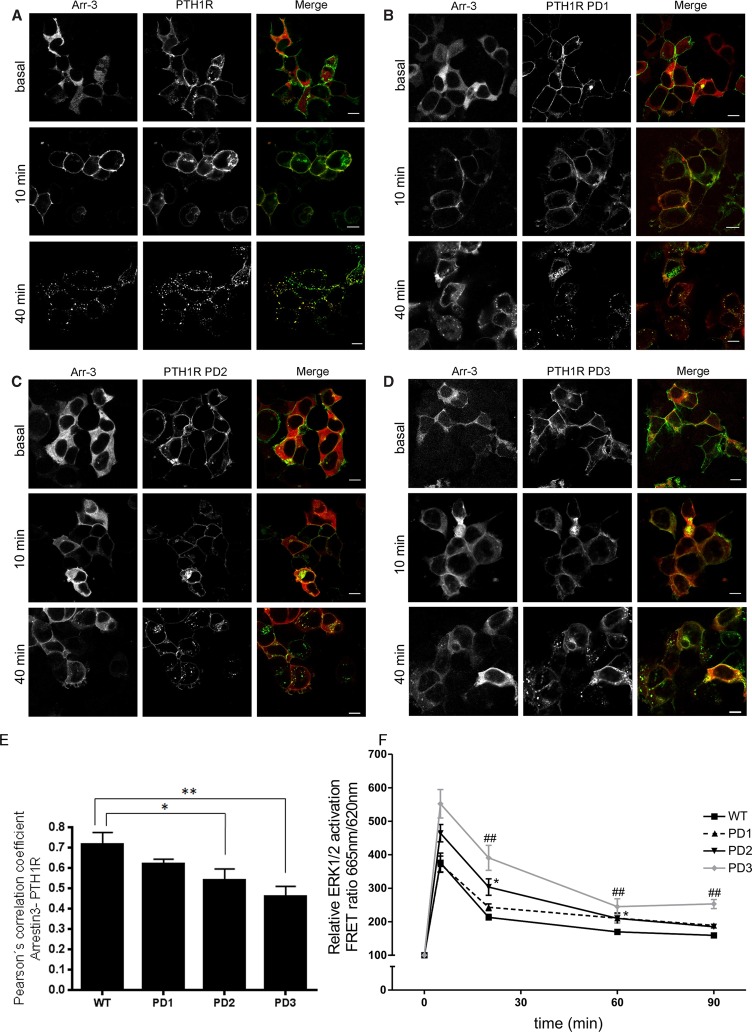
C-terminal phosphorylation sites determine arrestin3 co-internalization with activated PTH1R. (**A–D**) HEK293T cells were transiently transfected with the PTH1R or PD1, PD2 or PD3 mutants and arrestin3–CFP. Cells were not stimulated or treated for 10 or 40 min with 1 µM PTH(1–34) in 0.1% BSA/Tyrode's buffer at 37°C. Cells were subsequently fixed in 4% PFA and analyzed by confocal microscopy. The scale bar represents 10 µm. The extent of arrestin translocation, arrestin3–receptor colocalization and PTH1R internalization is substantially reduced in the PD3 mutant compared with WT. (**E**) Quantification of colocalization of PTH1R–YFP with arrestin3–CFP was performed using Pearson's correlation coefficient. Analysis was performed on samples following 40 min of stimulation with PTH(1–34). Mean data are presented *± *SEM; *n* ≥ 34 cells, ANOVA followed by Dunnett's multiple comparison test, differences between conditions are significant as indicated; *P < 0.05, **P < 0.01. (**F**) Activation of pERK1/2 was assessed in HEK293T cells expressing PTH1R or PD1, PD2 or PD3 mutants using an HTRF assay. Cells were starved overnight and stimulated with 500 nM PTH(1–34) in 0.1% BSA for the time shown. Data are representative of three independent experiments and are presented as the mean response ± SEM. Time points at which mutant PTH1R responses are significantly different from WT are indicated. Full statistics are described in Table 4.

In FRAP experiments, the arrestin exchange rate at the immobilized PTHR was low, suggesting a very strong interaction between arrestin3 and the agonist-activated WT PTH1R. PTH1R is known to co-internalize with arrestin upon agonist stimulation, and it has been previously suggested that the PTH1R C-terminus contains determinants of receptor internalization that are distinct from receptor phosphorylation [17]. Using HEK293T cells transiently transfected with CFP-tagged arrestin3 and eYFP-tagged PTH1R, we have investigated the impact of PTH1R C-terminal mutants on receptor internalization and arrestin trafficking. In resting cells, arrestin3 was localized within the cytoplasm and PTH1R was evenly distributed at the plasma membrane (Figure 7A, top panel). Stimulation with PTH(1–34) induced a translocation of arrestin from the cytosol to the plasma membrane in cells expressing WT PTH1R within 8–10 min. After 40 min of agonist exposure, PTH1R redistributed from the plasma membrane to become co-localized with arrestin3 in endocytic vesicles (Figure 7A, lower left). The high-affinity interaction observed between PTH1R PD1 and arrestin in FRAP experiments was also reflected in the translocation of arrestin and co-localization with the PD1 receptor, which after 40 min of stimulation appeared to be similar to WT PTH1R (Figure 7B). The co-localization of arrestin3 with the PD2 and the PD3 mutant was considerably reduced compared with the WT or the PD1 receptor mutant (Figure 7C,D, lower panels; Figure 7E), indicating very limited redistribution of these PTH1R mutants from their plasma membrane location particularly in the case of PTH1R PD3.

The activation of ERK1/2 is receptor-specific and cell-type-dependent. Using HTRF analysis in HEK293 cells, we have evaluated the effect of PTH1R, PD1, PD2 and PD3 on PTH(1–34)-induced ERK1/2 activation. Consistent with previous studies, PTH(1–34) stimulation of the WT receptor resulted in ERK1/2 phosphorylation, which peaked at 5 min [33–35] and did not fully reach the baseline even after 90 min (Figure 7F; Table 4). This was also the case for PTH1R PD1 whose ERK1/2 activation profile was not significantly different from WT. In contrast, the phosphorylation-deficient mutant PD2 exhibited an ERK1/2 activation profile, which was significantly exaggerated compared with WT PTH1R after 5 min of stimulation but no different at subsequent time points. The PD3 mutant exhibited an ERK1/2 activation profile that was both significantly exaggerated and prolonged compared with WT PTH1R, indicating that ERK1/2 phosphorylation was inversely correlated with ligand-dependent phosphorylation in cluster 2 of the PTH1R.

## Discussion

For many GPCRs, activation leads to rapid receptor phosphorylation, which in turn promotes the recruitment of arrestin leading to receptor internalization and the initiation of G-protein-independent signaling pathways [36]. Here, we focus on the PTH1R, which responds to the 84-amino-acid peptide hormone PTH and is essential to the physiology of calcium homeostasis and bone remodeling. In line with previous reports on the opossum PTH1R [14–16,37,38], we found the human PTH1R to be robustly phosphorylated in response to agonist stimulation. Using mass spectrometry phospho-proteomics, we have identified nine sites in the C-terminal tail between Ser473 and Thr551 (Table 1) and have subsequently characterized the role of these phosphorylation sites in arrestin recruitment, receptor internalization and ERK signaling.

Previous studies have described the phosphorylation profile of the opossum PTH1R receptor that has high sequence homology with human PTH1R. This receptor was found to be exclusively phosphorylated on C-terminal serine residues, and mutation of six serine residues (Ser483, Ser485, Ser486, Ser489, Ser495 and Ser498) to alanine abolished agonist-mediated phosphorylation altogether [37]. Here, our analysis has revealed phosphorylated residues distal to Ser498 and our findings agree, at least in part, with radiosequencing experiments performed on the rat PTH receptor which indicated that Ser491, Ser492, Ser493, Ser495, Ser501 and Ser504 were phosphorylated in response to stimulation with PTH [16]. Three of the phosphorylation sites that we identified in the PTH1R C-terminal tail have not been previously reported: Thr503, Ser519 and Thr547. Of these, Ser519 was repeatedly observed by MS/MS analysis, indicating that this site may be highly phosphorylated. Another, unexpected, phosphorylation site was identified in the extracellular N-terminus of the PTH1R at position Ser73. It remains to be determined whether this phosphorylation event occurs during PTH1R synthesis or whether phosphorylation of the N-terminus by extracellular kinases represents a novel mode of functional receptor regulation.

Targeted removal of the C-terminal phosphorylation sites revealed that the majority of agonist-regulated phosphorylation occurred within two clusters of residues: Ser489–Ser495 and Ser501–Thr506. Mutation of phosphorylation sites between Ser501 and Thr506 reduced agonist-regulated ^32^P incorporation by ∼25%, whereas mutation of the sites between Ser489 and Ser495 reduced it by nearly two-thirds. Analysis by FRET and BRET revealed that the contribution of these clusters to arrestin recruitment was not equal with cluster 2 having a greater impact on arrestin recruitment despite having a relatively small impact on agonist-induced phosphorylation. Our data indicate that arrestin3 is able to differentially recognize and interact with PTH1R variants displaying different phosphorylation profiles, but despite this the two phosphorylation clusters also act in concert to promote the formation of stable arrestin–receptor complexes. The sequential removal of phosphorylation clusters 1 and 2 produced a graded reduction in recruitment resulting in a receptor mutant (PD3), which recruited arrestin poorly compared with WT PTH1R and reduced receptor internalization and co-trafficking of receptor–arrestin complexes in response to stimulation with PTH(1–34). Surprisingly, this receptor mutant resembled the WT receptor in its ability to stimulate the production of cAMP through coupling to G_s_ proteins. The mutant PD3 did, however, display increased levels of constitutive activity compared with WT. The use of heterologous overexpression, which results in high receptor expression levels, might explain why in this system desensitization was not observed and an increase in PTH(1–34)-induced cAMP formation in the PD3 mutant, which might be expected due to its reduced interaction with arrestin, was not measured. We extended our study further by investigating the impact of PTH1R phosphorylation on the activation of the ERK1/2. Arrestins have been proposed to mediate a slow phase of GPCR-mediated ERK1/2 activation [39]. A phosphorylation- and arrestin-binding-deficient receptor should, therefore, show reduced ERK1/2 activation. However, mutant PD2 displayed a significantly enhanced and prolonged ERK1/2 activation, an effect which was further enhanced in mutant PD3, indicating that the ERK response observed here is not a consequence of arrestin recruitment. This is in line with a previous study in which mutated PTH1R phosphorylation sites were shown to prolong ERK1/2 activation in LLCP-K_1_ cells independently from internalization [33]. Similarly, shRNA silencing of arrestins has been shown to enhance ERK1/2 activation mediated by the α_2A_-adrenergic receptor and the sphingosine 1-phosphate receptor [40] and siRNA knockdown of both GRK and arrestin enhanced ERK1/2 activation by the M_3_-muscarinic acetylcholine receptor [41]. Taken together, these findings suggest that targeted removal of receptor phosphorylation sites results in a PTH1R which is uncoupled from arrestin recruitment and could therefore be considered biased towards the G-protein arm of GPCR signaling.

Further mutational analysis of phosphorylation sites located outside of clusters 1 and 2 at the proximal and distal end of the PTH1R C-tail revealed that these sites, either in isolation or in combination with mutations in clusters 1 and 2, had no impact on arrestin recruitment, suggesting that with regard to receptor phosphorylation, the important regulatory sites are located within these two clusters. More detailed mutational analysis of cluster 2 using FRET and FRAP identified a single threonine (Thr503) and a single serine (Ser504), which served as key determinants in modulating arrestin recruitment and the affinity of arrestin–receptor complexes. These findings are in agreement with previous studies of the opossum or the rat PTH1R [30,42] where phosphorylation sites in this region were reported to be important regulators of arrestin translocation, but here the important contributions specifically of Thr503 and Ser504 were overlooked. To some extent, PTH1R resembles the β_2_-adrenergic receptor (β_2_AR) and other GPCRs in so far as the proximal phosphorylation sites in the C-tail mediate full arrestin interaction [43]. Yet, whereas a mutant β_2_AR with a phosphorylation-deficient C-tail no longer interacts with arrestin, mutation of 18 of 26 potential phosphorylation sites in PTH1R, including all of those identified as being phosphorylated by MS/MS, creates a receptor which still interacts with arrestin in an agonist-dependent manner. This could potentially be due to the contributions of other ‘structural’ elements such as glutamate or aspartate residues within the C-tail, which could ‘mimic’ receptor phosphorylation and contribute to arrestin preactivation and recruitment as has been reported for free fatty acid receptor 4 (FFAR4) [26]. However, that this is not the case for PTH1R is indicated by experiments on a C-terminally truncated PTH1R, which still recruited arrestin in an agonist-dependent manner albeit to a much reduced extent compared with WT. This observation is also in agreement with previous studies on a C-terminally truncated PTH1R [17]. Studies on the M_2_-muscarinic receptors and β_2_ARs have demonstrated that at least in some cases the dependence on phosphorylation for recruitment of arrestin to GPCRs is not equal as a phosphorylation-deficient M_2_-muscarininc receptor interacts with arrestin normally, whereas arrestin recruitment to a phosphorylation-deficient β_2_AR was substantially reduced [44]. In this way, PTH1R could be considered to display an intermediate phenotype as receptor phosphorylation is necessary for establishing stable high-affinity complexes, but is not essential for promoting receptor:arrestin interaction. FRAP experiments demonstrate that in contrast with the β_2_AR, the PTH1R forms complexes with arrestin3 that are not dissociable at the time scale of the experiment. However, the marked differences in the kinetics and the extent of arrestin3 recovery observed with the phosphorylation-deficient mutants revealed that the second cluster, in particular residues Ser503 and Thr504, are responsible for the majority of the high-affinity interaction between arrestin and the PTH1R. Hence, the temporal stability of arrestin3–PTH1R complexes are determined by the localization of specific phosphorylation sites within the PTH1R C-tail.

The standard model of arrestin activation involves not only the phosphate sensor in the core of arrestin but also the activation sensor that recognizes the activated receptor [24,45]. Recently, detailed structural studies have reported interactions between the arrestin ‘finger loop’ and elements of the activated receptor core including elements within transmembrane helices VII and VIII [46,47]. It is conceivable, therefore, that PTH1R activation contributes to the preactivation of arrestin, and the phosphorylation and C-terminus-independent arrestin recruitment observed here is the result of a low-affinity arrestin–receptor interaction possibly mediated by the arrestin ‘finger loop’.

Previous work from our laboratory and other groups has highlighted the importance of the positioning of receptor phosphorylation in relation to arrestin recruitment and receptor function. Patterns of receptor phosphorylation have been determined for receptors such as FFAR4, the ghrelin receptor (GHSR1a), chemokine receptor CXCR4, the β_2_AR and the M_3_-muscarinic receptor [26,31,32,48], and it would appear at least in these instances that receptor phosphorylation represents more than just the addition of bulk negative charge to a certain receptor domain. Indeed, the position of phosphorylation sites within the receptor C-terminus or intracellular loop regions appears to be key to regulating arrestin recruitment and receptor function. This certainly appears to be the case for PTH1R as phosphorylation sites within cluster 1 have a smaller impact on arrestin recruitment, suggesting that phosphorylation within cluster 1 may perform a modulatory or stabilizing role possibly fine-tuning arrestin–receptor interactions as has been suggested for other receptors [32]. In contrast, phosphorylation within cluster 2 is responsible for establishing the high-affinity receptor–arrestin interaction. These data would support a notion that has been suggested for other receptors that a phosphorylation signature or barcode adopted by activated GPCRs can determine the receptor signaling outcome [22]. Given that different tissue and cell types express different complements of kinases at different levels and that evidence of cell-type-specific regulation of GPCR signaling already exists [31], the possible physiological outcomes of this are that PTH1R signaling output might be tailored by the cell type in which the receptor is expressed.

In summary, a combination of [^32^P]orthophosphate labeling studies and mass spectrometry has been used to comprehensively analyze PTH1R phosphorylation. Two clusters of serine and threonine residues have been identified within the proximal C-terminal tail, which modulates potency, efficacy and affinity of arrestin3 binding. Mutation of these two clusters of phosphorylation sites resulted in a substantial reduction in arrestin binding when assessed by BRET and FRET methods and resulted in a mutant that displayed hallmarks of a receptor that is biased away from arrestin signaling and toward G-protein signaling. Complete abolition of arrestin interaction was, however, not achieved even after truncation of the receptor C-tail immediately after helix 8, findings which are in strong agreement with published results. It is now clear that a combination of mutations of C-tail phosphorylation site and further mutations probably within helices VII and VIII would be necessary to fully abolish PTH1R arrestin recruitment as was the case for the recently reported mutant, G-protein-biased M_3_-muscarinic receptor [49]. Work is underway to generate such a PTH1R mutant that would be a valuable tool for the investigation of the cellular signaling profile with respect to G-proteins and arrestin; this in turn would inform the search for PTH1R ligands that could direct receptor signaling along one pathway in preference to another.

## Abbreviations

ANOVA: analysis of variance
β_2_AR: β_2_-adrenergic receptor
BRET: bioluminescence resonance energy transfer
cAMP: cyclic adenosine monophosphate
CFP: cyan fluorescent protein
CMV: cytomegalovirus
DMEM: Dulbecco's modified Eagle's medium
ELISA: enzyme-linked immunosorbent assay
ERK1/2: extracellular-signal-regulated kinase 1/2
FBS: fetal bovine serum
FFAR4: free fatty acid receptor 4
FRAP: fluorescence recovery after photobleaching
FRET: fluorescence resonance energy transfer
GPCR: G-protein-coupled receptor
GRK: G-protein-coupled receptor kinase
HA: haemagglutinin
HEK: human embryonic kidney
HRP: horseradish peroxidase
HTRF: homogeneous time-resolved fluorescence
IL: interleukin
IP_3_: inositol 1,4,5-trisphosphate
MS/MS: tandem mass spectrometry
NHERF2: Na^+^/H^+^ exchanger regulatory factor 2
PDZ: post synaptic density protein (PSD95) Drosophila disc large tumor suppressor (Dlg1) zonula occludens-1 protein (zo-1)
PFA: paraformaldehyde
PKA: protein kinase A
PLC: phospholipase C
PTH: parathyroid hormone
PTHR: parathyroid hormone receptor
Rluc: *Renilla* luciferase
S/T: serine/threonine
WT: wild-type
YFP: yellow fluorescent protein.
